# The Evolution of Robin Sequence Treatment Based on the Biomimetic Interdisciplinary Approach: A Historical Review

**DOI:** 10.3390/biomimetics8070536

**Published:** 2023-11-10

**Authors:** Martin Čverha, Ivan Varga, Tereza Trenčanská, Barbora Šufliarsky, Andrej Thurzo

**Affiliations:** 1 Clinic of Pediatric Otorhinolaryngology of the Medical Faculty Comenius University in Bratislava and National Institute of Children’s Diseases, 83101 Bratislava, Slovakia; tereza.trencanska@nudch.eu; 2 Institute of Histology and Embryology, Faculty of Medicine, Comenius University in Bratislava, 81372 Bratislava, Slovakia; ivan.varga@fmed.uniba.sk; 3 Department of Oral and Maxillofacial Surgery, Faculty of Medicine, Comenius University in Bratislava and University Hospital, 81372 Bratislava, Slovakia; barbora.sufliarsky@fmed.uniba.sk; 4 Department of Orthodontics, Regenerative and Forensic Dentistry, Faculty of Medicine, Comenius University in Bratislava, 81102 Bratislava, Slovakia

**Keywords:** Pierre Robin sequence, micrognathia, personalized, appliance, biocompatible, glossoptosis, regenerative dentistry, 3D printing

## Abstract

The Robin sequence is a congenital anomaly characterized by a triad of features: micrognathia, glossoptosis, and airway obstruction. This comprehensive historical review maps the evolution of approaches and appliances for its treatment from the past to the current modern possibilities of an interdisciplinary combination of modern engineering, medicine, materials, and computer science combined approach with emphasis on designing appliances inspired by nature and individual human anatomy. Current biomimetic designs are clinically applied, resulting in appliances that are more efficient, comfortable, sustainable, and safer than legacy traditional designs. This review maps the treatment modalities that have been used for patients with a Robin sequence over the years. Early management of the Robin sequence focused primarily on airway maintenance and feeding support, while current management strategies involve both nonsurgical and surgical interventions and biomimetic biocompatible personalized appliances. The goal of this paper was to provide a review of the evolution of management strategies for patients with the Robin sequence that led to the current interdisciplinary biomimetic approaches impacting the future of Robin Sequence treatment with biomimetics at the forefront.

## 1. Introduction

The Robin sequence (RS) was described in 1923 by Pierre Robin [1], a French stomatologist. The triad of micrognathia, glossoptosis, and airway obstruction was initially thought to be a disorder separate from a cleft palate. However, it is now known that RS is often associated with a cleft palate and is a subset of the larger spectrum of craniofacial anomalies. Since its initial description, management of RS has evolved significantly, with advances in both surgical and nonsurgical treatments [2]. This congenital condition impacts a relatively small proportion of infants, with an estimated incidence ranging from 1 in 8000 to 1 in 14,000, as reported by Maas and Poets in 2014 [2]. However, a more recent study conducted by Wright et al. suggests a higher prevalence, exceeding 1 in 5000 infants [3]. Due to the close association of the described symptomatology with Robin’s publications, most experts addressing neonatal micrognathia from the 1960s onwards adopted the term Pierre Robin syndrome [4].

## 2. Overview of Diagnostic Approaches and Understanding the Pathology

In the first recorded instance within medical literature Fairbairn (1846) [5] documented the case of a newborn who had a cleft palate, posterior positioning of the lower jaw and difficulties with proper breathing and feeding. This ultimately resulted in the infant’s death within two days. During the autopsy, it was discovered that a “thick and short” tongue was obstructing the pharynx and larynx, with no other pathological findings present.

In 1923, Pierre Robin [1] reported on patients with mandibular hypoplasia who had undergone one or more adenoidectomies but failed to experience any improvement. He postulated that the pathophysiological mechanism underlying upper airway obstruction (UAO) could be attributed to occlusion occurring at the junction of the base of the tongue and the epiglottis. Furthermore, he put forward the hypothesis that individuals with congenital “glossoptosis”, a term he introduced, exhibited more profound mental disorders.

In 1950, Douglas [6] delineated the mechanism of airway collapse linked with micrognathia, which he referred to as “linguo-epiglottic obstruction”. Through laryngoscopic observations, he discovered that the tongue would fall backward and downward in the pharynx, exerting pressure on the epiglottis in a manner that generated a ball valve-like mechanism. This, in turn, permitted the outflow of air while impeding its inflow.

In current literature, the focus is aimed at improving the quality of life, but in former times, a diagnosis of RS was often fatal for the majority of infants. According to Robin’s report in 1934 [7], none of the infants with severe retrognathia had survived beyond 18 months. He wrote in his conclusion, “I have never seen a child live more than 16 to 18 months who presented hypoplasia such as the lower maxilla was pushed more than 1 cm behind the upper”. Later, in 1950, Douglas [6] reported a mortality rate of 65% in 21 patients treated nonsurgically.

According to Kiskadden and Dietrich’s (1953) [8] findings, out of 15 patients treated only by positioning, mortality was reported in 33%, and 60% developed aspiration pneumonia. The 10 patients who survived using positioning alone experienced minimal weight gain, with 9 failing to put on any weight during the first two months. Most of the patients exhibited gradual weight gain in the first year of life.

Airway obstruction in RS is commonly described as developing soon after birth [9], but its onset can also be delayed [10]. In a study of 10 patients with mandibular catch-up growth-related UAO, seven cases occurred between 24 and 51 days of age [11].

In severe cases of RS, the child may exhibit inspiratory stridor, increased respiratory effort, and even apnea and cyanosis. Severe respiratory compromise is typically demonstrated by carbon dioxide retention and hypoxemia. However, mild glossoptosis, which is common in individuals with RS, may not induce these symptoms, but can increase the risk of sleep-disordered breathing [12].

In recent years, there has been a shift towards using polysomnography to assess an infant’s breathing, which is much more sensitive when documenting upper airway obstruction and its resulting hypoxia. This was documented in several studies, including those by Freed et al. [13], Bull et al. [14], and Gilhooly et al. [15]. Intermittent UAO may occur more frequently during sleep, whereupon polysomnography provides valuable information [12].

Airway endoscopy has proved to be a valuable tool, particularly for identifying the root cause of obstruction, as confirmed via polysomnography [16]. RS exhibit a broad spectrum of airway obstruction, with varying severity. Some cases are straightforward to diagnose, while others are more complex. In some unclear cases, when nasopharyngolaryngoscopy is performed on children that are awake, their agitation can lead to muscular tension, affecting the procedure’s accuracy [12]. To address this issue, drug-induced sleep endoscopy may be employed, uncovering hidden obstructions, as suggested by Lee et al. [17] in their study [12,16,17]. Another serious condition connected with RS is dysphagia. The safe and efficacious ingestion of food relies on a series of intricately coordinated, sequential movements. Specifically, the act of sucking precedes that of swallowing, which is periodically interrupted by breathing. In individuals with RS, dysphagia can arise due to several factors. Firstly, a palatal defect may lead to regurgitation of the food bolus, as well as an inability to generate negative pressure during the sucking process. Secondly, the retrograde positioning of the tongue can obstruct the upper airway and weaken the suction mechanism, further contributing to dysphagia. In the context of syndromic RS, the patient’s mental or neurological impairment may also impact their nutritional status [18]. According to de Vries et al. (2013) [19], there are a range of feeding difficulties caused by RS that can be classified into several categories, including decreased oral intake, hazardous oral feeding, protracted feeding durations, and the emergence of respiratory complications. 

To create an interdisciplinary consensus, a meeting of 145 specialists from 24 different countries was held in Utrecht, The Netherlands, in 2014. An expert panel was assembled with the objective of formulating an RS clinical consensus report, which was developed through the synthesis of existing literature and expert opinions. During the discussion, six panels were held to address the following topics: micrognathia, glossoptosis, airway obstruction, feeding difficulties, cleft palate, and etiology. As a result, the following triad is mandatory for an RS diagnosis: UAO, glossoptosis, and micrognathia. RS can occur as an isolated condition or as a part of syndrome or multiple-anomaly disorder [12]. 

In a study conducted by Holder-Espinasse et al. (2001) [20], which examined 117 individuals diagnosed with RS, the authors found that 48% had isolated RS, 35% had RS associated with an identifiable syndrome, and 17% had RS where an unidentified syndrome was suspected as the cause. A genetic analysis of 125 RS patients conducted by Izumi et al. (2012) [21] revealed associated syndromes in 58% of cases, with Stickler and Marshall syndromes being the most prevalent. A recent study by Stoll et al. [22] found an even higher incidence of associated anomalies in RS cases (69.7%) compared to isolated RS (30.3%). In the syndromic RS group, the authors identified various conditions: 11.2% of the patients had chromosomal abnormalities, 30.3% had syndromic conditions not associated with chromosomal anomalies, and 28.1% exhibited multiple congenital anomalies, notably affecting the ear, face and neck, cardiovascular, neurological, urinary, and ocular systems.

It is recommended that all children with suspected RS undergo a comprehensive assessment by a clinical geneticist, as some syndromes may not become apparent until later in life.

In addition to micrognathia, there are several methods for evaluation, including low-dose multisection CT, cone beam CT, lateral cephalogram, plaster casts, 3-D photography, and direct measurements using rulers and calipers.

However, the diagnosis of micrognathia can be subjective for most clinicians, as there is currently no gold standard for objectively evaluating this sign. The jaw index score is a tool that meets the requirements of availability, reproducibility, cost-effectiveness, and safety for evaluating micrognathia. In addition to micrognathia, many individuals with RS also develop maxillary hypoplasia, as noted by Breugem et al. (2016) [12]. Cephalometric analysis is standard method to identify micrognathia with historically two significant paradigm shifts represented by the transition from manual to digital cephalometric analysis [23] and recently by the paradigm shift caused by AI-driven cephalometric analysis [24,25,26].

### The Embryological Basis and Etiopathogenesis of Robin Sequence

The embryological basis of the Robin Sequence is not fully understood, but it is thought to be caused by a combination of genetic and environmental factors. During early embryonic development, the regions of the face and anterior neck are derived from transient embryonic pharyngeal (branchial) arches. From evolutionary point of view, these arches represent a region in which the development of gills during the ontogenesis of all chordates has been recapitulated [27]. The first and second pharyngeal arches collectively make up the facial skeleton, the viscerocranium. Although all three embryonic germ layers (the ectoderm, mesoderm, and endoderm) come together to assemble the pharyngeal arches, most of the tissue within viscerocranial skeletal components differentiates from the neural crest derived cells. The neural crest is a vertebrate-specific migratory population of multipotent stem cells that originate in the region between the neural and non-neural ectoderm, during the process of formation of neural tube—the primordium of the future central nervous system. The multipotent cell population of neural crest cells contributes to proper development of the mesenchyme of the pharyngeal region: the future muscles, cartilages, bones, and connective tissues of the face, including the tongue [28,29]. Failure of neural crest development can cause a variety of pathologies, often syndromic, that are globally called neurocristopathies [30,31]. A typical example of neurocristopathy is the Robin sequence. Many genes are known to be involved in neural crest development, but not all of them have been identified. Mutations of genes regulating and/or disrupting the signaling pathway of migration, proliferation, and/or differentiation of neural crest cells may be the genetic cause of the human Robin sequence and highlight the interconnection of palate, tongue, and mandible embryonic development [32].

Numerous hypotheses have been proposed to explain the etiopathogenesis of isolated RS. It is generally agreed that this condition represents a sequence of pathological processes initiated by micrognathia, which leads to glossoptosis, potentially resulting in airway obstruction and cleft palate. Abadie et al. [33] have suggested a potential etiology involving intrauterine and neonatal brainstem dysfunction. Another theory [34] related to tongue development points to a delay in lingual neuromuscular development. Furthermore, there is a straightforward hypothesis implicating external compression of the mandible, which can be caused by oligohydramnios, uterine anomalies, or multifetal pregnancy.

## 3. Treatment Modalities

### 3.1. Nonsurgical Treatment

#### 3.1.1. Positioning

The first recorded successful therapeutic approach to treating UAO was positioning. Accurately positioning the head and upper body can provide some level of relief. Robin (1923) [1] was the first to report on the practice of orthostatic feeding, whereby patients were positioned during feeding in a prone position to move their chin forward. This technique works by shifting the tongue forward, preventing it from descending into the hypopharynx and thereby improving breathing [35].

In 1980, Lewis and Pashayan [36] proposed a technique involving the prone position and a large nasogastric tube to stent the tongue forward and prevent the base of the tongue from being fully compressed against the posterior pharyngeal wall in infants with RS. Later Pashayan and Lewis (1984) [37] published a case series detailing their management of 25 RS infants using this method and the results showed no incidence of fatalities.

While positional treatment has been reported to yield success rates of 40–70% for RS infants with UAO [38], its effectiveness as a long-term solution remains limited [39]. Based on a retrospective study evaluating the effectiveness of a prone sleep position for obstructive sleep apnea (OSA) in RS patients, Coutier et al. (2019) [40] concluded that this positioning technique does improve OSA symptoms. However, the study found that a prone sleep position was only sufficient to decrease the OSA index below the severe level in 3 out of 18 patients studied, suggesting that it may not be an effective treatment option for all RS patients. Additionally, authors found that a third of patients did not present clinical manifestations of UAO despite having severe OSA, as confirmed by polysomnography. This highlights the importance of using objective measures to diagnose OSA in infants with RS, even if they do not show overt clinical symptoms.

**Figure 1:**
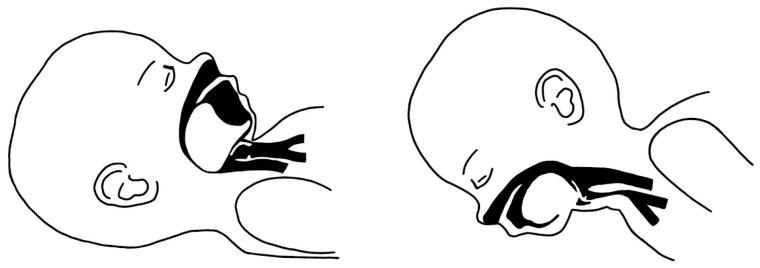
Patency of the upper airways is achieved via prone positioning.

There is scarce investigation into the effectiveness of positional treatment based on sleep study results, and current available results are not in favor of using the prone position as a single treatment modality. Moreover, the prone position is linked to a heightened risk of sudden infant death syndrome [41]. Patency of the upper airways is achieved via prone positioning—Figure 1.

#### 3.1.2. Stenting

Another straightforward method for maintaining patent upper airways in RS patients is using tubes inserted into the upper airways. Axtrup (1963) [42] first described the use of a nasopharyngeal airway (NPA) as a treatment to relieve respiratory distress in RS infants. Initially, it was used for mild cases of obstruction or as a temporary relief before surgical intervention [4,43]. During the late 1990s, NPA became a preferred treatment modality due to their demonstrated effectiveness. This was evidenced in research conducted by Masters et al. (1999) [44]. In a study by Wagener [45], 22 infants with RS were exclusively treated with a NPA, with an average usage of 44 days and an average hospital stay of 60 days. Sixteen infants were partially fed through a nasogastric tube and partially orally.

Pediatric NPA are utilized to maintain the airway in neonates, but issues with tube fixation can arise due to slight changes in position, which can lead to a loss of airway control and oxygen desaturation. Furthermore, the adhesive tapes employed to attach the NPA to the face often become wet and detach, compromising the airway. To address these challenges, Smyth (1998) constructed a simple acrylic splint to securely hold the NPA in place, thereby maintaining the airway and facilitating feeding. The author recommended using these splints for a duration of 10 days to 4 weeks while closely monitoring the patient through sleep studies.

In 2015, a study was conducted by Drago Marquezini Salmen & Lazarini Marques (2015) [46], which involved 223 patients with RS infants. Of these patients, 73% had an isolated form of the condition while 27% had the syndromic form. Among the patients, 107 were diagnosed with severe respiratory difficulty and Type I and Type II according to Sher classification. Subsequently, these patients received nasopharyngeal intubation. On average, the duration of tube use was 57 days, and the patients stayed in the hospital for an average of 18 days. Notably, all patients were able to avoid a tracheostomy or were successfully decannulated. A total of 15% of patients required a gastrostomy, but there were no recorded instances of mortality. 

**Figure 2:**
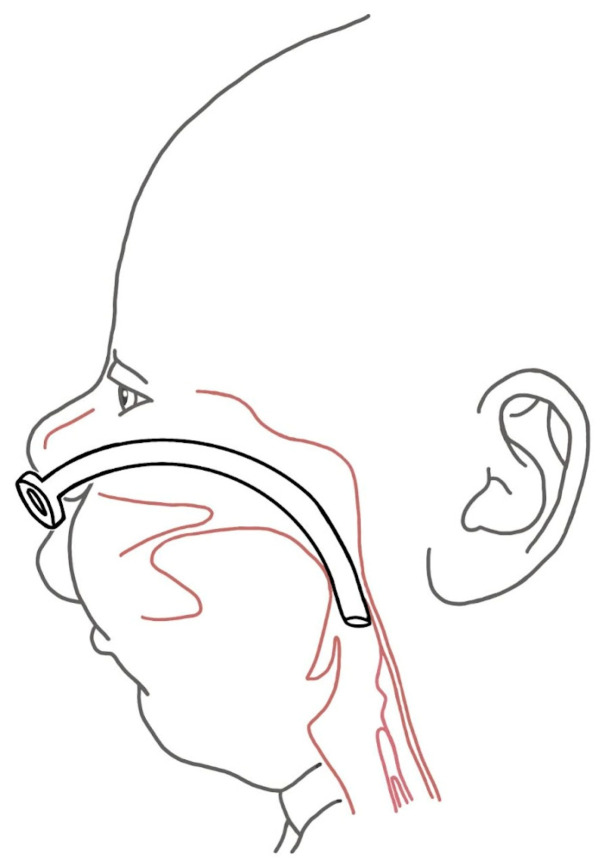
The maintenance of the airway patency by nasopharyngeal airway.

Some experts consider the nasopharyngeal tube (Figure 2) as one of the most revolutionary options for managing respiratory difficulty [35]. However, despite its proven efficacy and minimal or no impact on morbidity and mortality, its use is not yet widespread. 

#### 3.1.3. Appliances

The evolution of technology has brought about a wide range of technical solutions that have progressed from being initially unpopular and cumbersome to becoming highly innovative and successful modes of treatment. 

In 1930, Eley & Farber (1930) [47] employed a head cap that was attached to a copper wire. This wire ran adjacent to the ramus and body of the mandible and under the chin, effectively holding the mandible in an anterior position. Nisenson (1948) [48] made modifications to this device; however, it remained cumbersome and difficult to use. As a result, it did not achieve widespread acceptance in the medical community.

**Figure 3:**
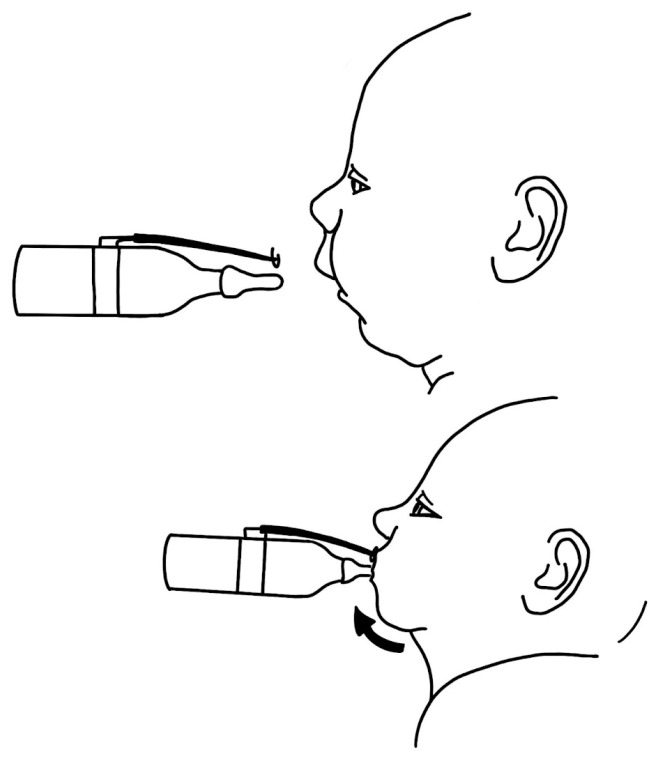
The “lip guard” feeding device introduced by Davis and Dunn forced the infant to thrust the lower jaw forward.

In 1933, Davis and Dunn proposed a hypothesis for stimulating mandibular growth in infants. They suggested the use of a bottle with a “lip guard” (Figure 3), which would require the infant to move their mandible forward to achieve proper lip closure while feeding.

In 1953, Kiskadden and Dietrich [8] introduced a pulley mechanism that applied forward traction on the jaw and tongue by fastening it to the bed. 

**Figure 4:**
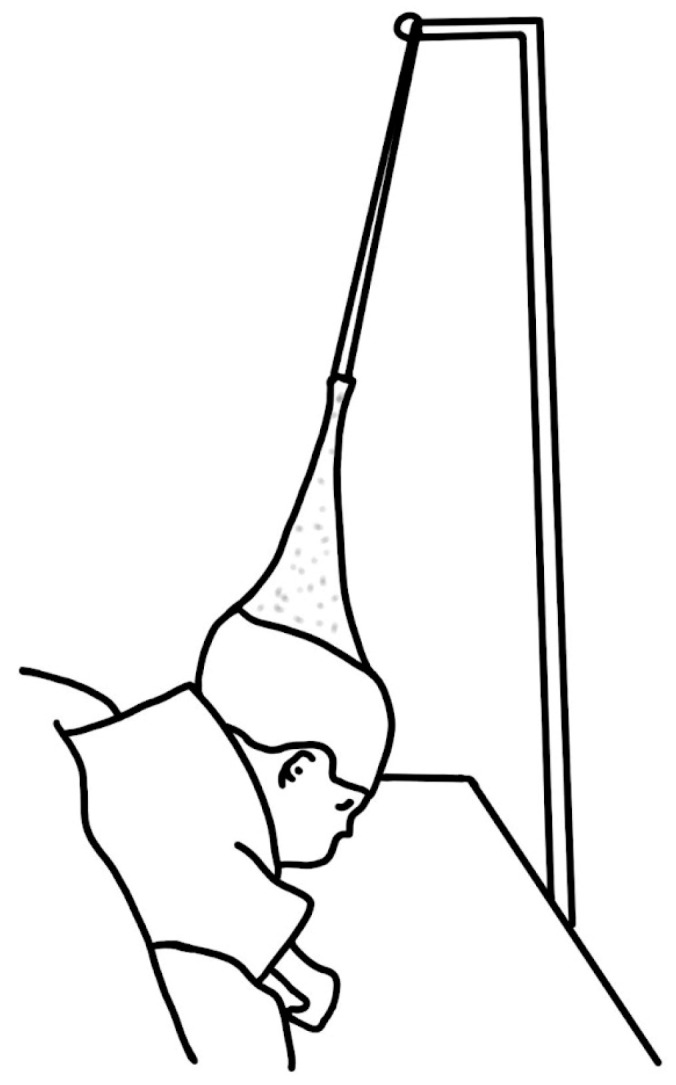
The principle of the device introduced by Dennison (1965) [9] was to improve the simple prone position.

Dennison [9] developed an “elastoplast cap” suspension device that pulled the head supero-posteriorly to the desired position. However, its benefits compared to tracheostomy are highly questionable (Figure 4).

Hotz and Gnoinski (1982) [49] recommended the use of intraoral orthopedic plates to obturate the cleft, thereby preventing the tongue from retracting into the cleft, which was believed to cause airway obstruction in individuals with RS.

**Figure 5:**
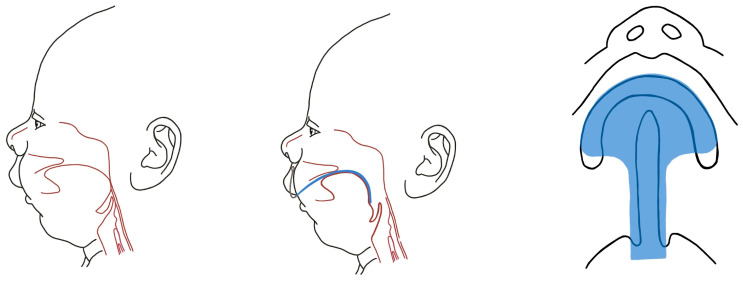
Acrylic palatal plate simultaneously alleviates the upper airway by retracting the tongue and obturates cleft palate, which enables sufficient physiological breathing and feeding.

In 1967, Pielou [50] introduced a successful treatment for RS using an acrylic obturator with a disto-palatal extension that can hold the tongue in an anterior position. These appliances, which are called palatal plates, are still successfully used in Tübingen [51]. The Tübingen Palatal Plate (TPP) is a highly effective functional orthodontic treatment for upper airway obstruction in infants with Robin Sequence (RS) [52,53,54,55]. Additive Manufacturing is frequently used to manufacture these appliances [56,57]. Three-dimensional printing in oral and maxillofacial surgery is now common practice [58] and use of biocompatible materials with biomimetic properties is the current trend in regenerative dentistry, among other applications [59]. Acrylic palatal plate simultaneously alleviates the upper airway by retracting the tongue and obturates cleft palate—Figure 5.

A similar device called the pre-epiglottic baton plate consists of an acrylic plate connected by an iron wire with velar extension made of distinct, colored hard acrylic. The palatal plate is designed to mimic the shape of the dentoalveolar process and hard palate, while the second, smaller plate, known as the “spur”, is oriented dorso-caudally and holds the tongue in an anterior position to maintain patency of the upper airway. Additionally, the palatal part can serve to obturate a frequently occurring cleft palate [60].

According to Buchenau et al. (2017) [55], over 33% of infants born with RS in Germany are administered a pre-epiglottic baton, and the use of this appliance resulted in a significantly reduced the frequency of mixed and obstructive apneas during sleep, as well as improving feeding problems and failure to thrive.

Poets et al.’s (2017) [61] prospective cohort study across three centers found that treatment with a pre-epiglottic baton plate resulted in significant improvement in breathing, as confirmed via polysomnography. Additionally, the treatment led to improved feeding, as evidenced by weight gain, and a significant decrease in patients relying on nasogastric tube nutrition.

There is evidence to suggest that these appliances can also accelerate mandibular growth, as demonstrated in a recent study by Effert et al. (2023) [62].

No severe adverse events such as bleeding, systemic infections, or aspiration were observed during the treatment with these appliances. The most common side effect reported was the development of tender spots on the hard or soft palate. However, all affected infants experienced improvement after manual reshaping of the plate, as reported by Buchenau et al. (2017) [55].

The Children’s Hospital Stanford in Palo Alto was the first institution in the United States to introduce an orthodontic airway plate. Their innovative approach involved creating an advanced plate based on the original Tubingen palatal plate design, but with a split expansion mechanism that allows for expansion in the lateral dimension and enables the plate to “grow” with the patient’s upper jaw. With this treatment, patients were able to complete the process with just one appliance [63]. Despite being deemed highly successful and safe by its proponents, this method has not gained more widespread acceptance. This may be attributed to the fact that it requires a multidisciplinary team approach, including upper airway endoscopists, sleep specialists, and a dental laboratory capable of manufacturing such a device, according to the authors.

Thurzo et al. (2022) [64,65,66] have proposed several solutions, including a new manufacturing concept for intraoral and extraoral parts of these appliances using 3D scanning and printing technologies. This development has the potential to make this mode of treatment more affordable and readily available on a global scale.

### 3.2. Surgical Treatment

#### 3.2.1. Tracheostomy

Before the advent of specialized surgical techniques for individuals with RS, tracheostomies were frequently employed to ensure airway patency. Nowadays, a tracheostomy is typically reserved as a last option when other interventions tailored to infants with RS prove ineffective. Multiple authors considered tracheostomy to be a “solution of defeat” [67,68]. This procedure comes with both short- and long-term complications, making it an undesirable option. The need for constant tracheostomy care, disruption of normal family life and parent–child bonding, and negative effects on speech development are considered significant drawbacks of early tracheostomy. In addition to the disadvantages previously mentioned, it is important to note that tracheostomy does not offer an entirely fail-safe airway solution, as demonstrated by a systematic review of tracheostomy in childhood. The review revealed a mortality rate of up to 6%, which was primarily attributed to issues such as cannula obstruction or accidental decannulation [69].

#### 3.2.2. Tongue-Lip Adhesion

Due to the unwieldiness, maintenance challenges, unhygienic nature, and frequent breakdowns of the mechanical devices used in the past, as well as the described drawbacks of a tracheostomy, alternative treatment options were sought. While there is literature mentioning tongue–lip surgical adhesion described by Shukowsky as early as 1902 [70], Douglas [6] is considered by many as a pioneer of this procedure in RS. He introduced glossopexy as a potential solution to this pathology. This surgical intervention, also known as tongue–lip adhesion (TLA), involved exposing a rectangular area of the floor of the mouth, extending from the alveolus to the lower lip. The tongue was then advanced, and the raw area beneath it was sutured across the exposed regions of the floor of the mouth, alveolus, and lower lip. Additionally, a mattress tension suture was placed from the tongue base to the chin, passing through the lower lip.

Singer and Sidoti (1992) [68] proposed a protocol for performing glossopexy, which involves an endoscopic evaluation to ensure the suitability of this procedure. Before surgery, they considered it necessary to wait for patient stabilization and attainment of some weight gain. The author suggests that gastrostomy should only be a last resort, as all patients with normal neurological status, except for one syndromic case, were able to successfully restore oral feeding.

Also, Sher (1992) [39] reported that glossopexy did not interfere with deglutition, and instead facilitated oral intake by providing an adequate airway.

Over the years, this technique has been widely used and modified [70,71]. However, despite its popularity, the potential for significant complications is a considerable burden associated with this procedure. Many surgeons have expressed dissatisfaction due to the complications that arise as a result of this procedure, such as dehiscences, tongue lacerations, wound infections, and scarring of the lip, chin, and floor of the mouth, as reported by several groups [36,71,72].

To address these concerns, Parsons [73] and Argamaso [43] state the significance of tongue muscular disinsertion from the mandible, which not only helps to correct the tongue position, but also reduces the risk of dehiscence.

**Figure 6:**
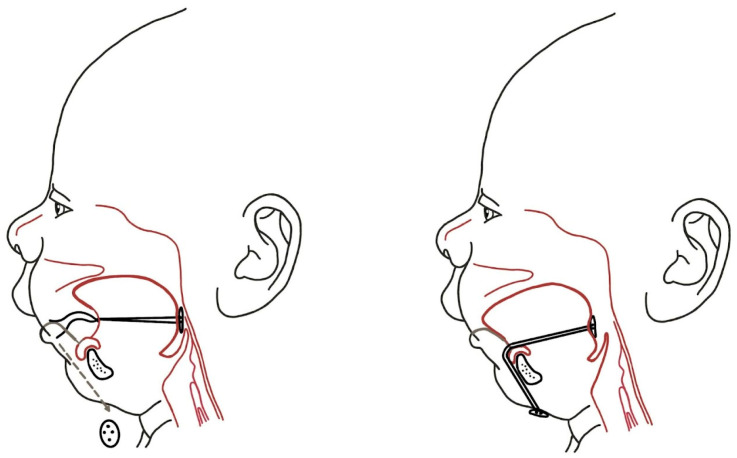
The principle of glossopexy also called tongue-lip adhesion is retraction of the tongue base by suture along with exposure of corresponding submucosal tissue of the tongue and lip to fusion.

According to Caouette-laberge et al. (1995) [38], the subperiosteal release of the floor of the mouth has been shown to be a successful treatment approach on its own. The authors suggest that the cause of tongue malposition is not posterior displacement, but rather posterior rotation of the tongue on its base due to the genioglossus muscle’s tight attachment to the mandibular symphysis. In their experience, after releasing the genioglossus, geniohyoid, and mylohyoid muscles, the tongue’s adjusted position is sufficient for proper breathing within 5–6 days. Figure 6 shows tongue-lip adhesion.

#### 3.2.3. Mandibular Distraction Osteosynthesis

TLA was the most effective surgical approach for correcting glossoptosis and respiratory obstruction prior to the introduction of distraction osteogenesis [74,75].

**Figure 7:**
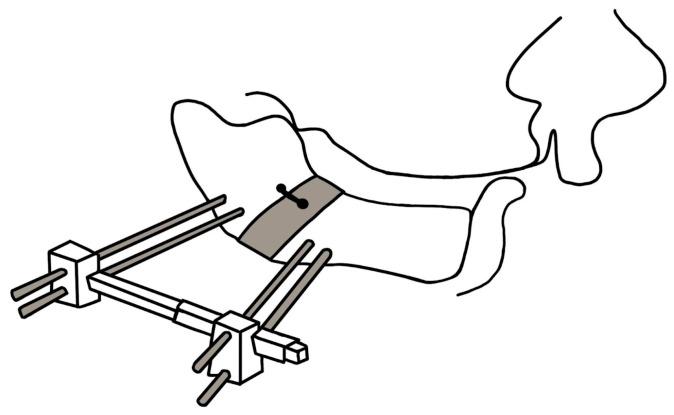
Mandibular distraction osteosynthesis is surgical method that elongates the mandible.

Cohen et al. (1998) [76] described the use of acute mandibular distraction with external distraction devices after bilateral osteotomy as a means of achieving improvements in breathing in UAO. The technique of mandibular distraction osteogenesis (MDO) utilizes progressive lengthening of the mandible to correct tongue ptosis, increase the size of the pharyngeal airway, and correct micrognathia, ultimately resulting in the elimination of UAO [77,78]. Cohen et al. (1998) [76] reported a complication in which there was an early loss of pin fixation, leading to the risk of relapse and mandibular displacement. Multiple authors considered total anesthesia as a relative disadvantage, and potential complications included iatrogenic damage to the inferior alveolar nerves and dental follicles, infection, mucosal perforation, bone formation defects, possible disturbance of intrinsic mandibular growth, as well as accompanying facial scarring. In their study, Denny et al. (2001) reported on six patients with RS sequence who were presented with obstructive symptoms that were successfully resolved following MDO. MDO is shown on Figure 7.

#### 3.2.4. Other Surgical Procedures

**Figure 8:**
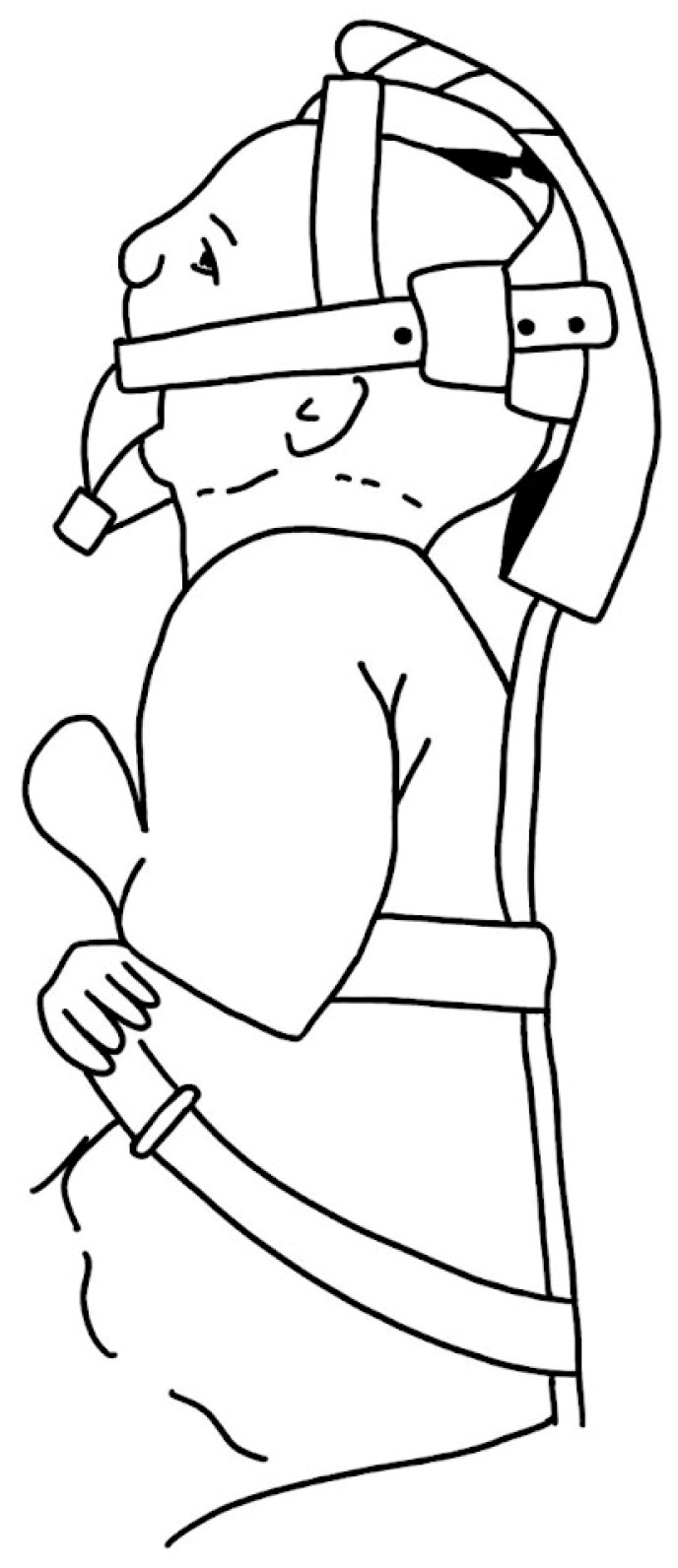
Cumbersome device advocated by Callister (1937) [79].

Aside from the commonly used TLA and MDO procedures, several lesser-known surgical approaches have been proposed to address UAO. Callister et al. (1937) [79] employed a thick silver wire encircling the mandibular symphysis, which was subsequently fastened to a brace device to facilitate forward traction (Figure 8).

Champion [80] suggested that palatal clefts should be repaired within the first 48 h of life based on the assumption that the tongue may become lodged in the palatal cleft, leading to nasopharyngeal obstruction.

Hadley and Johnson (1963) [81] successfully treated a patient with RS by passing a Kirchner wire through both mandible bodies and the radix of the tongue. 

In 1968, Lewis et al. [82] employed a one-stage procedure in which they utilized a fascia lata graft to create a sling that could be used to bring the tongue into approximation with the mandible. However, the authors did not provide any commentary regarding the potential impact of this procedure on oral feeding. 

Lapidot et al. [83] reported using a stainless-steel wire to wrap around the hyoid bone for the purpose of stabilizing the tongue in an anterior position.

**Figure 9:**
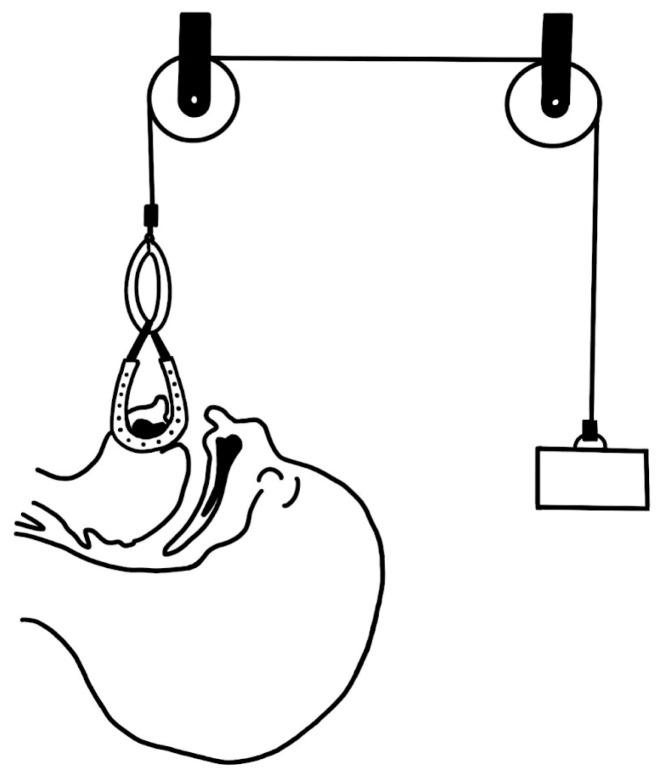
Pulley system proposed by Wada et al. (1983) [84].

Tongue traction (Figure 9) has been observed to be largely ineffective due to the sutures used for retraction frequently pulling through the lingual muscle before the child develops sufficient strength, or before the jaw can grow to accommodate the tongue in an unobstructed position [43]. In the 1980s, Wada et al. [84] published a paper about mandibular traction device. This method is still in use in some centers and it continues to be a topic of discussion in the literature [85,86].

Hyomandibulopexy is another surgical technique used to reposition the tongue in RS patients; however, it has not gained the same level of popularity as glossopexy [43]. During this procedure, the hyoid bone is anchored to the mandible anteriorly, making it more difficult to visualize and intubate the larynx. Furthermore, this technique may interfere with the growth potential of the mandible [38].

At present, there is a paucity of research comparing the advantages and disadvantages of the surgical techniques currently in use. Bekisz et al. [87] conducted a review of the literature and found no studies that assessed other surgical outcomes related to cranial reconstruction for craniosynostosis, Le Fort III type distraction/advancement, monobloc distraction/advancement, hypertelorism reconstruction, or surgical outcomes related to the treatment of patient populations affected by RS.

In a retrospective study conducted by Flores et al. [75], which compared the experience of a single surgeon with TLA and MDO, the results demonstrated that MDO yielded superior outcome measures in terms of oxygen saturation, apnea–hypopnea index, and the incidence of tracheostomy. Although both procedures were associated with complications, the TLA had a higher rate of complications.

Viezel-Mathieu et al. (2016) [88] reported contradictory findings. In their systematic review, they identified a cohort of 268 patients with RS. Among the various treatment modalities assessed, TLA was found to be effective in alleviating airway obstruction in 81.3% of cases, with lower complication rates (13.8%) compared to MDO (23.8%) and tracheostomy (37.5%).

## 4. Discussion

Airway obstruction resulting from micrognathia and glossoptosis is the most significant symptom of RS. As a result, treatment focuses on mandibular or tongue advancement to alleviate these symptoms. Additionally, effective feeding management is an important aspect of treatment. Biomimetic principles offer a promising new approach to developing treatments for airway obstruction in RS. For example, a biomimetic design of oral appliances could be used to keep the tongue in place and prevent airway obstruction. By applying biomimetic principles, new and improved treatments that can enhance the quality of life for patients with RS can be developed.

Currently, first-line nonsurgical interventions for obstructive sleep apnea in RS include prone sleep [40] and the use of supplemental oxygen. Due to ethical considerations, it is not permissible to conduct surgical procedures for the purpose of comparing their effectiveness with prone positioning in patients who show a favorable response to the latter. Therefore, most of the studies examining the management of respiratory obstruction in patients with RS had a low and very low quality of evidence, primarily due to their methodological design. Nonetheless, several studies have shown that prone positioning can be an effective initial measure for managing respiratory obstruction in RS patients, with success rates ranging from 41% to 69% [35].

Based on the findings of Wilson et al. (2000) [11], which report a delayed development of UAO in RS patients, a safer conservative option emerges in the form of an application of an oropharyngeal or nasopharyngeal tube [89], noninvasive ventilation (typically continuous positive airway pressure), and oral appliances with velar extension [61]. Although CPAP is a widely used method for UAO, due to the nature of isolated RS, the use of a nasopharyngeal airway and a pre-epiglottic baton plate, which are smaller and easier to manipulate, is preferred. These two methods have been shown to effectively facilitate physiological breathing and feeding with minimal to no impact on morbidity and mortality. However, despite their effectiveness, these techniques are not yet widely used, and surgical strategies remain the first-line management in many institutions around the world. Nowadays, surgical options include TLA, MDO, subperiosteal release of the floor of the mouth, and tracheostomy.

As indicated by the literature, there is no comprehensive comparison of the available therapeutic options. However, it is possible to conduct a study to evaluate the effectiveness of nasopharyngeal tubes and pre-epiglottic baton plates, which have recorded a high success rate with negligible complications. In addition to the pre-epiglottic baton plate, there is evidence supporting the hypothesis of stimulation of mandibular catch-up [62], which was previously reached exclusively by MDO. These two noninvasive methods fulfill requirements for physiologic breathing and feeding, require only simple maintenance from parents, and allow secure and relatively early discharge from medical facilities. These factors contribute to the development of a healthy relationship between the infant and parents and make management of this serious condition as nontraumatic as possible. Another argument supporting noninvasive treatment is that by the age of 6 months, most RS patients, regardless of treatment, naturally outgrow these difficulties [10]. It should be noted that while NPA and oral appliances can be effective, they also have several drawbacks. One major drawback is the need for continuous care provided by parents throughout the entire treatment process, as not all caregivers are capable of providing this. Additionally, there is a risk that the appliance may not be inserted properly or may become damaged, which could lead to potential risks associated with respiratory distress, particularly if such an incident occurs during the night. However, the risk of a fatal incident is minimal when care is provided by well-educated and cooperating parents, and with the assistance of a wide range of breathing monitors, pulse oximeters, and other technical devices for monitoring the child’s condition. The second drawback, especially for oral appliances, lies in the requirement for skilled personnel and advanced equipment, particularly in the production of intricate devices like the pre-epiglottic baton plate. However, this challenge may be mitigated by leveraging innovative technologies and automation [64].

While surgical methods may appear to be a straightforward solution, they come with a host of potential disadvantages. Take, for instance, procedures like TLA or MDO, which often require multiple sessions and entail all the risks associated with surgical intervention and general anesthesia. Furthermore, each type of surgery poses unique risks that need to be considered. Equally significant is the psychosocial dimension. Surgery can be a source of anxiety and apprehension for parents, who may worry about their child’s well-being and the implications of the intervention. On the other hand, after a successful surgery, the post-operative care for a child with RS is akin to that of a healthy child.

As noted by Gómez et al. (2018) [35], there is currently no consensus in the literature regarding the treatment of patients with RS. Furthermore, there is a lack of multicenter studies comparing different management modalities for this condition. Resnick et al. in 2019 [90] conducted a survey to gather expert opinions on the diagnosis and treatment of patients with RS. The survey included a substantial number of craniofacial surgeons and non-surgeon physicians. The results showed that for patients with obstructive hypopnea/apnea, 53% of experts would recommend nasogastric tube insertion, 47% would recommend nasopharyngeal airway insertion, 44% would recommend surgical procedures, and only 11% would recommend CPAP. Experts from the United States were more likely to recommend MDO (82%) compared to non-US experts, who had similar rates of recommending TLA, MDO, tracheostomy, and other procedures. US experts also tended to recommend earlier surgical intervention.

A new guideline for the diagnosis and treatment of RS has recently been released. The guideline, developed by the European Reference Network for rare and/or complex craniofacial anomalies and ear, nose, and throat disorders (ERN-CRANIO), is a comprehensive resource for healthcare professionals who care for patients with Robin Sequence. The guideline covers all aspects of RS, from diagnosis and management to long-term care. It is based on the latest evidence and expert consensus and provides recommendations for the best practices in the care of these patients. The publication of this guideline is a significant milestone in the effort to establish high standards of care for patients with Robin Sequence. It is also a valuable resource for healthcare professionals and patients alike [91].

The selection of a specific treatment plan often depends on the severity of airway obstruction, the presence of accompanying anomalies, and the medical team’s preferences and expertise in managing the patient. In general, it can be stated that conservative therapy typically requires a more prolonged and sustained effort from both the parents and a broader interdisciplinary team. Conversely, in the case of a surgical procedure, the substantial responsibility for achieving success primarily falls on the surgical team. It is necessary to say that conservative and surgical methods are not mutually exclusive, but on the contrary, they complement each other. Understandably, the best results of RS treatment are achieved by multidisciplinary teams capable of providing all the described modalities according to the needs and possibilities of the patient and his parents. Integral members of these multidisciplinary teams include pediatricians, neonatologists, orthodontists, dental surgeons, otolaryngologists (ENT doctors), speech therapists, sleep medicine specialists, clinical geneticists, plastic surgeons, pediatric anesthesiologists, and other healthcare professionals [92].

The most suitable determinants for attaining the optimal treatment modality are the physiological requirements of the patients.

The approach of conservative treatment has historically been associated with numerous limitations. However, with the advent of contemporary digital and technological advancements, these constraints can be readily surmounted, as we exemplify in this review. The cumbersome devices have evolved into user-friendly appliances through innovation and design enhancements. Adhering to the anatomical proportions and dynamics of individual muscular, osseous, and mucosal structures offers a relatively precise delineation of an “ideal device”. So-called palatal plates, as elucidated by several authors [60,61,63,64], conform to various requisites stipulated for this “ideal device”. These appliances replicate the distinct anatomical characteristics of individual patients, facilitating near-physiological dynamics of both breathing and feeding reflexes. The challenge associated with the extensive adoption of these devices lies in the requisite involvement of a diverse multidisciplinary team and the relatively intricate fabrication process required for each device. We maintain the belief that a broad spectrum of innovations exists that is capable of enhancing individual patient comfort and facilitating the dissemination of this treatment modality to various regions worldwide. The capacity to digitize an individual’s anatomical structures presents an opportunity for remote collaboration among multiple teams. Access to 3D printing technology and the availability of biocompatible materials result in the simplification and reduction of fabrication time. This modality also broadens the range of potential materials that can be utilized, facilitates the creation of intricate shapes, and enables the seamless reproduction of designed models with desired modifications, thereby unlocking untapped potential for enhancements. Our vision entails the development of a flexible device, a hybrid amalgamation of a palatal plate and an airway stent, obviating the need for external attachments. Ideally, this device would eliminate any intricate post-processing steps, such as the addition and bending of wires, and offer a seamless and effective solution.

New concepts of advanced regenerative dentistry have the potential to revolutionize the treatment of RS by making it possible to develop new surgical interventions that focus on tissue regeneration. These often include biomimetic hydroxyapatite materials and 3D printing or bioprinting, thus opening up new perspectives for surgical approach in respect to hard tissue regeneration [93,94,95]. However, the future surgical interventions in RS would depend not only on bone, but especially on soft tissue adaptations, for which perspectives are significantly improving with findings of Danišovič et al. that mesenchymal stromal cells (MSCs) from bone marrow, adipose tissue, and umbilical cord have similar biological properties and chondrogenic potential, making them all promising candidates for cartilage tissue engineering [96]. The fact that MSCs can be obtained from a variety of sources, including bone marrow, adipose tissue, and umbilical cord, makes them a versatile and accessible cell therapy option. This is particularly important for RS patients, as they often have difficulty with traditional surgical interventions due to the complexity of their disease.

The evolution of management strategies for patients with Robin sequence historically led to the current interdisciplinary biomimetic approaches. Biomimetic principles are inspired by nature and can be applied to a wide range of fields, including medicine and engineering. In the context of Robin sequence, biomimetic principles could be used to develop new and improved nonsurgical interventions. In the past, the crowd-sourcing mechanism—comparing various designs and materials from a wide pool of different researchers—was typical modus operandi in advancements on shape, material, and other appliance properties in RS therapy [97]. Today, the implementation of machine learning and wider applications of advanced AI presents a new, accelerated way of evaluating effective appliance designs and biomimetic material properties that will soon provide astonishing results [98]. Researchers from Massachusetts Institute of Technology recently developed a generative-AI-driven tool that enables the user to add custom design elements to 3D models without compromising the functionality of the fabricated objects. A designer could utilize this tool, which is called Style2Fab, to personalize 3D models of objects using only natural language prompts to describe their desired design. The user could then fabricate the objects with a 3D printer [99]. It is only a matter of time until AI tools will support doctors in 3D designing, personalization and improving efficiency of individualized appliances for biocompatible 3D printing.

Another biomimetic principle could also be used to develop new surgical techniques of RS therapy that are less invasive and more effective; for example, a surgical technique that uses a patient’s own stem cells to create new bone and tissue. Stem cell-based surgery, according to this review, shows the potential of development of such a surgical technique that uses the patient’s own stem cells to create new bone and tissue. This technique could be used to correct the underlying skeletal abnormalities that cause Robin sequence.

Recently, various promising methods were introduced that could improve biomimetic perspectives of 3D-printed biocompatible appliances. For example, the zinc-containing coatings can improve appliance surfaces, boosting its antibacterial properties against various strains of bacteria [100]. Various antimicrobial biomaterials, with not yet fully understood antimicrobial mechanisms, have been introduced as potential material for removable 3D-printed appliances for RS therapy. “Researchers gradually found that welcoming microbial cellular adhesion to a lethal surface was a more effective solution than targeting microbial cellular repulsion when designing antimicrobial surfaces”. Xiang Ge, 2019 [101]. Finite Element Analysis is now a standard method for researching the most favorable biomechanical shape for various implantable as well as removable appliances [102,103], which helps to reduce stress levels in surrounding living tissues.

By applying biomimetic principles to the development of new nonsurgical interventions for Robin sequence, we could improve the quality of life of patients with this condition.

## 5. Conclusions

The treatment of RS has evolved considerably over the last century, with advances in both surgical and nonsurgical treatments. Historical development shows an inclination towards designs and materials taken from nature, with the trend towards increasing interdisciplinary collaborations in engineering, chemistry, medicine, and biology applied to the synthesis of practical materials and appliance designs that can mimic the structure, function, and shape of native biological systems. While early treatment strategies focused on simple securing of airway maintenance and feeding support, today’s treatment strategies encompass a multidisciplinary, complex approach that includes both nonsurgical and surgical interventions to ensure the most physiological development possible for an infant with RS. As technology advances, the treatment of RS is likely to evolve and improve, further incorporating current trends in biomimetics.

## Data Availability

Not applicable.
